# Thaumatin-like Gene *TLP1b* Confers to Seed Oil Content and Resistance to *Sclerotinia sclerotiorum* in Arabidopsis

**DOI:** 10.3390/ijms26051930

**Published:** 2025-02-24

**Authors:** Jinghang Liao, Shucheng Qi, Hong Huang, Hongmei Liao, Yixin Cui, Zhi Liu, Wei Qian, Hongli Dong

**Affiliations:** 1 Integrative Science Center of Germplasm Creation in Western China (CHONGQING) Science City, College of Agronomy and Biotechnology, Southwest University, Chongqing 400715, China; liaojinghang_cm@163.com (J.L.); qsc1052362467@163.com (S.Q.); hwanghong95@163.com (H.H.); liaohongmei502x@163.com (H.L.); cuiyx1992@163.com (Y.C.); liuzhi84@swu.edu.cn (Z.L.); 2 Engineering Research Center of South Upland Agriculture, Ministry of Education, Chongqing 400715, China

**Keywords:** *AtTLPb*, Arabidopsis, circadian rhythm, *S. sclerotiorum*, seed oil content

## Abstract

The synergistic optimization of yield and abiotic/biotic resistance is of great significance in plant breeding. However, the genomic mechanisms underlying the selection for environmental adaptation and yield-related traits remain poorly understood. In this study, we identified a thaumatin-like protein (TLP), AtTLP1b, which was shown to pleiotropically regulate seed oil content and resistance to *Sclerotinia sclerotiorum* by gene knockout and overexpressing experiments in Arabidopsis. The oil composition oleic acid (C18:1), linoleic acid (C18:2), linolenic acid (C18:3) and eicosenoic acid (C20:1) were altered significantly in overexpressing and knockout lines. RNA-seq analysis revealed that overexpression of *AtTLP1b* significantly downregulated the expression levels of genes involved in wax, suberin synthesis, oxylipin metabolism and plant–pathogen interaction. Furthermore, more than half of the genes involved in the circadian rhythm–plant pathway were differentially expressed in the overexpressing lines. We propose that *AtTLP1b* primarily inhibits fatty acid synthesis and plant immune responses via the circadian rhythm–plant pathway. Our findings suggest that *AtTLP1b* plays a vital role in simultaneous improvement of seed oil content and resistance to *S. sclerotiorum* and provides a valuable resource for molecular breeding.

## 1. Introduction

Vegetable oil serves as a major edible oil and a renewable energy product globally [1]. Food and energy demand is steadily increasing due to rapid growth in the global population and commodity consumption rates. It is estimated that global agricultural production will need to be increased by at least 60–100% by 2050 to meet these demands [2]. Therefore, increasing oil production by breeding high-yield oil crops is a key objective for breeders. Oil production in seeds begins with de novo fatty acid (FA) biosynthesis and elongation, followed by the incorporation of these FAs into triacylglycerol (TAG) biosynthesis [3]. Arabidopsis acyl-lipid metabolism represents a complex biochemical network, encompassing a minimum of 120 enzymatic reactions and involving more than 600 genes that encode the associated proteins and regulatory factors. These pathways are categorized into 16 sections based on the types of lipids produced and their subcellular localization [3].

In our previous study, we identified several hub genes that potentially regulate seed oil content through gene co-expression analysis [4]. A thaumatin-like gene *AtTLP1b* that has high connectivity is co-expressed with several known regulators of seed oil content in the co-expression network [4]. Thaumatin-like proteins (TLPs) are a highly complex protein family associated with host defense and developmental processes in plants, animals, and fungi [5]. They are highly diverse in angiosperms, for which they are classified as the Pathogenesis-Related-5 (PR-5) protein family, a family of proteins with high sequence similarity to thaumatin which is a sweet-tasting protein from the West African shrub *Thaumatococcus danielli* [6]. The analysis of the conserved domain organization of TLPs shows that the thaumatin domain (Pfam: PF00314) covers almost 95% of the entire mature peptide [7]. TLPs generally possess 16-cysteines residues, forming eight disulfide bonds that contribute to their structural stability [6]. Most of the cloned TLPs have been demonstrated to play important roles in biotic or abiotic stresses. *Thau1/2/3/4* from Arabidopsis functions in both biotic stress and abiotic stress signal transduction through the regulation of proline synthesis [8]. *GhTLP1* responds to abscisic acid (ABA) and jasmonic acid (JA) hormones, and enhances cotton resistance to *Verticillium dahliae* through the MAPK signaling pathway-plant [9]. Recombinant expression of *TaTLP2-B* in *Saccharomyces cerevisiae* confers significant tolerance against cold, heat, osmotic, and salt stresses in bread wheat [10]. In addition, several TLPs are implicated in physiological and developmental processes. Membrane localized thaumatin-like protein from tea (CsTLP) enhances seed yield and the plant survival under drought stress in Arabidopsis [11]. Currently, there is no experimental evidence demonstrating the involvement of TLPs in the regulation of seed oil content or fatty acid biosynthesis and accumulation processes.

FAs and their derivatives play important roles in responding to biotic and abiotic stresses. For example, linolenic acid (C18:3) is involved in protein modification in plants under heat stress [12]. The levels of oleic acid (C18:1) and linoleic acid (C18:2) regulate, to some extent, the development, colonization, and mycotoxin production of fungi such as *Aspergillus* spp. [13,14]. In present study, we aimed to investigate the gene function of a thaumatin-like gene *AtTLP1b* on seed oil content and resistance to *S. sclerotiorum*, and attempted to elucidate the relationship between them. These findings will enhance our understanding of the relationship between seed oil content and resistance to *S. sclerotiorum* and provide a pleiotropic gene for genetic engineering breeding.

## 2. Results

### 2.1. In Silico Analysis and Subcellular Localization Analysi of AtTLP1b

**Figure 1:**
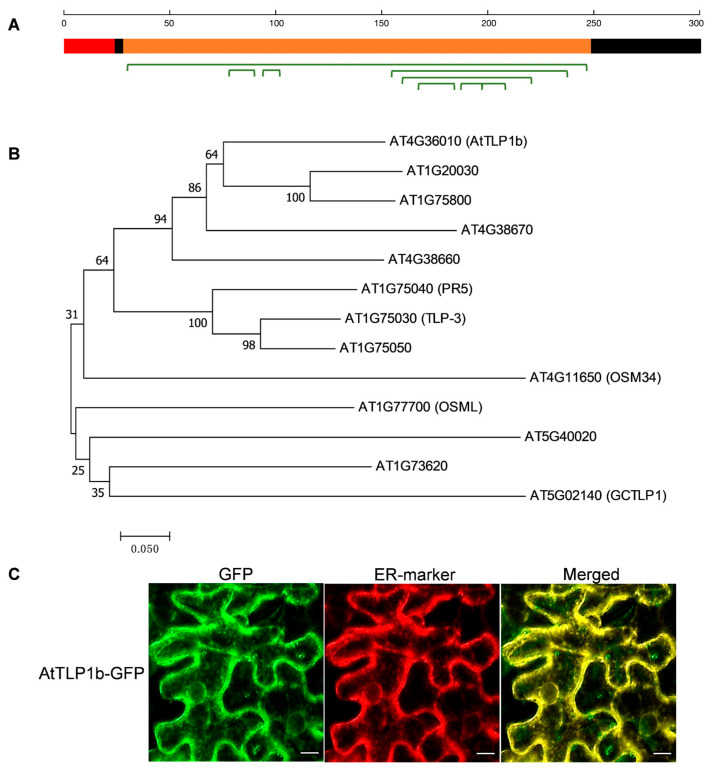
AtTLP1b is a typical TLP localized at the ER. (**A**) Schematic diagram of AtTLP1b protein structure. The red and orange boxes represent the signal peptide and the thaumatin domain of AtTLP1b.The black boxes represent protein without structural features. The green brackets represent the disulfide bonds. (**B**) Phylogenetic tree analysis of the TLP family in Arabidopsis was conducted using 13 amino acid sequences. The evolutionary distances were calculated, and the optimal tree with a total branch length of 3.40385098 was constructed. The tree is scaled, with branch lengths reflecting the evolutionary distances used for phylogenetic inference. Gaps and missing data were excluded from the analysis, resulting in a final dataset comprising 207 aligned positions. (**C**) From left to right, GFP represents green fluorescence under confocal microscopy, and ER-marker was used to visualize ER, which exhibited red fluorescence under confocal microscopy. Merged indicates the overlap of GFP channel and ER-marker channel, showing yellow fluorescence under confocal microscope. Scale bars = 10 μm.

*AT4G36010* encodes a pathogenesis-related thaumatin-like protein. We cloned the cDNA and genomic DNA sequences of *AT4G36010* using RT-PCR and PCR, respectively. It consists of 301 aa with a calculated molecular weight of 30.91 kDa (NCBI GenBank Accession No. NP_195325) (Figure 1A). The thaumatin domain (Pfam: PF00314) covers from 29-250aa (Figure 1A). A signal peptide was identified at the N-terminal of the predicted polypeptide sequence, with a predicted cleavage site between the 24th and 25th residues (Figure 1A). The Uniprot program (http://www.uniprot.org) predicted that AT4G36010 has 16 strictly conserved cysteine residues, which form 8 disulfide bridges at the position 31–249, 79–90, 95–102, 156–239, 161–222, 169–185, 189–198, and 199–209 of amino acid sequence (Figure 1A). These results suggest that AT4G36010 belongs to TLP family; therefore, we named this gene *AtTLP1b* according to its homologous rapeseed gene *BnaA01.TLP1b*, which encodes a thaumatin-like protein 1b. The Arabidopsis genome contains 12 homologous proteins within the same TLP family as AtTLP1b. Protein sequence alignment revealed that the TLP family did contain 16 strictly conserved cysteine residues (Figure S1). Subsequently, the phylogenetic tree of TLP family in Arabidopsis was constructed, and the results showed that AtTLP1b was most closely related to AT1G20030 and AT1G75800 (Figure 1B). However, these two genes have not been extensively studied on genetic functions.

To detect the subcellular localization of AtTLP1b, the *Agrobacterium tumefaciens*-containing AtTLP1b-GFP fusion construct was injected into tobacco leaves for transient expression. The endoplasmic reticulum (ER)-marker pTRAkc-RFP-his6-ERH was co-expressed to visualize ER. Green fluorescence in AtTLP1b-GFP transfected tobacco cells was confined to the ER and co-localized with the red fluorescence of the ER-marker, indicating that AtTLP1b-GFP is an ER localized protein (Figure 1C).

### 2.2. AtTLP1b Is a Negative Regulator of Seed Oil Content in Arabidopsis

**Figure 2:**
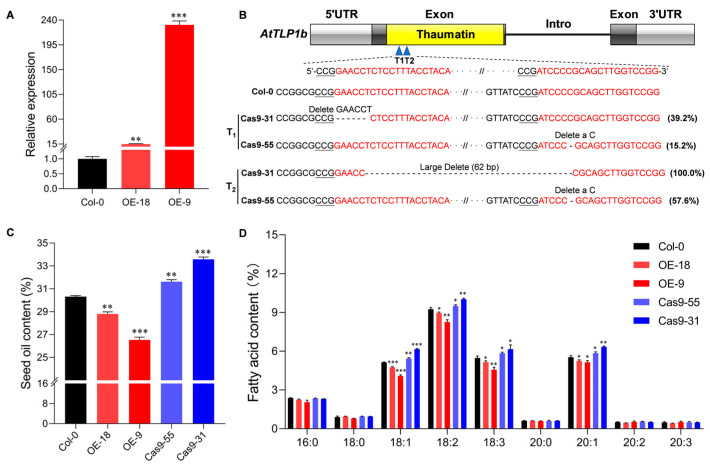
Gene expression analysis and the seed oil content of the overexpressing and knockout lines of *AtTLP1b* in *A. thaliana*. (**A**) Relative gene expression of the overexpressing lines. Three technical duplicates were used for RT-qPCR analysis. (**B**) The selection of sgRNA for the knockout vector and the editing types of T_1_ and T_2_ generations of knockout lines. The gray box represents 5′UTR or 3′UTR, the black box represents exons, and the yellow box represents thaumatin domain, and the black straight line represents intron. The blue triangle represents two sgRNA target sites, the red text represents the sgRNA target sequence, and the underlined text is the PAM sequence “NGG”. (**C**) The seed oil content of overexpressing lines and knockout lines of *AtTLP1b*. Three biological repetitions were used for analysis. (**D**) The content of each fatty acid component. The asterisk represented significant differences between *AtTLP1b* overexpressing lines/knockout lines and Col-0 (* *p* < 0.01, ** *p* < 0.01, *** *p* < 0.001. Student’s *t*-test).

To investigate the function of *AtTLP1b* in seed oil content, a transformation vector containing the coding region of *AtTLP1b* under the control of constitutive cauliflower mosaic virus (CaMV) 35S promoter was constructed and used to transform Arabidopsis Columbia ecotype (Col-0). Finally, two homozygous overexpressing lines (OE-9 and OE-18) in T_3_ generation along with Col-0 were selected for gene expression and seed oil content analysis. RT-qPCR analysis revealed that the transcript level of *AtTLP1b* in OE-18 and OE-9 was significantly increased than that in Col-0 (Figure 2A). Furthermore, we constructed loss-of-function mutants of *AtTLP1b* based on CRISPR-Cas9 system. Two small guide RNAs (sgRNAs) were designed within the conserved thaumatin domain of *AtTLP1b* and cloned into the pCas9-TPC-Red vector (Figure 2B, Table S1). In T_1_ generation, we found two high-probability edited lines (Cas9-31 and Cas9-55). Cas9-31 carried a 6 bp deletion at the first sgRNA target site, and Cas9-55 carried a 1 bp deletion at the second sgRNA target site (Figure 2B). In T_2_ generation, the 6 bp deletion in Cas9-31 became a large fragment deletion (62 bp) between the two target sites, the deletion in Cas9-55 was the same as in the T_1_ generation, with an increased frequency (Figure 2B).

Correspondingly, the average seed oil contents of OE-18 and OE-9 were, respectively, about 28.8% and 26.5%, which was significantly decreased by 5% and 12.5% compared to Col-0 (Figure 2C). In contrast, the average seed oil contents of Cas9-55 and Cas9-31 were, respectively, about 31.6% and 33.6%, which were significantly increased by 4.3% and 10.8% compared to Col-0 (Figure 2C). These results suggested that *AtTLP1b* negatively regulated seed oil content in Arabidopsis seeds. The detailed FA composition analysis showed that C18:1, C18:2, C18:3 and eicosenoic acid (C20:1), were the four major FAs in the seeds (Figure 2D), accounting for over 80% [15]. Compared to Col-0, the content of C18:1, C18:2, C18:3 and C20:1 was significantly decreased in overexpressing lines OE-18 and OE-9, while significantly increased in knockout lines Cas9-55 and Cas9-31 (Figure 2D). These results indicated that *AtTLP1b* altered the major FAs in Arabidopsis seeds.

### 2.3. AtTLP1b Negatively Regulated the Resistance to S. sclerotiorum

**Figure 3:**
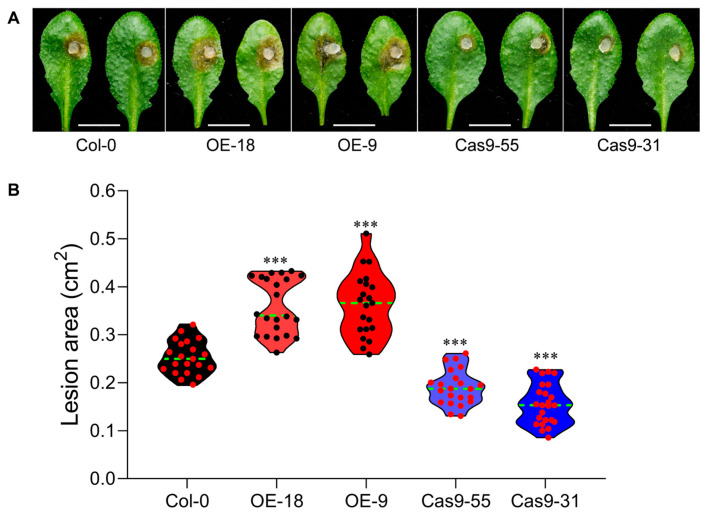
Overexpression and knockout of *AtTLP1b* affect the resistance to *S. sclerotiorum* in Arabidopsis. (**A**) The overexpressing lines and knockout lines of *AtTLP1b* along with Col-0 were challenged with *S. sclerotiorum* strain 1980. Scale bars = 1 cm. Photographs were taken at 24 hpi. (**B**) Statistical analysis of the lesion area induced by 1980 on overexpressing lines and knockout lines of *AtTLP1b* together with Col-0 at 24 hpi. Each small red/black dot represented a measured value, the green dotted line represented the median, and the asterisk represented significant differences between *AtTLP1b* overexpressing lines/knockout lines and Col-0 (*** *p* < 0.001. Student’s *t*-test).

It has been reported that TLPs may be involved in antifungal immune responses [9]. To explore initial insights of *AtTLP1b* in pathogen resistance, overexpressing and knockout lines of *AtTLP1b* were inoculated with *S. sclerotiorum*, which is a necrotrophic filamentous fungal pathogen that attacks more than 408 species of plants, especially for *Cruciferous* plants [16]. After 24 h post inoculation (hpi), the lesion areas of the overexpressing lines OE-18 and OE-9 were significantly larger than that of Col-0 (Figure 3A). The average lesion area of OE-18 and OE-9 was both about 0.36 cm^2^ (Figure 3B). In contrast, the average lesion area of Col-0 was about 0.25 cm^2^ (Figure 3B). Moreover, the average lesion area of Cas9-55 and Cas9-31 was, respectively, about 0.19 cm^2^ and 0.16 cm^2^ (Figure 3A,B). The knockout lines Cas9-55 and Cas9-31 exhibited significantly enhanced the resistance to *S. sclerotiorum* compared to Col-0. These results suggested that *AtTLP1b* negatively regulates the resistance to *S. sclerotiorum* in Arabidopsis.

### 2.4. Most Acyl-Lipid Metabolism (ALM) Genes Are Related to the Synthesis of Very-Long-Chain Fatty Acid Derivatives

In order to investigate the possible role of *AtTLP1b* in seed oil content and the resistance to *S. sclerotiorum*, RNA sequencing on siliques of OE-9, OE-18 and Col-0 lines collected at 4 days after flowering (DAF) (A4, B4, C4), 8 DAF (A8, B8, C8), 12 DAF (A12, B12, C12), 16 DAF (A16, B16, C16) was performed using Illumina HiSeq 2500 platform. A total of 79.18 Gb clean data were obtained after mRNA sequencing for 12 samples with at least 6.16 Gb clean data for each sample. In each sample, the bases score Q30 exceeded 93.25% (Table S2). The clean data were then mapped to the reference genome, with the mapping ratio varying from 96.00% to 97.65% (Table S2). Compared to Col-0, 862, 1362, 899 and 1786 overlapping DEGs were identified in two overexpressing lines of 4 DAF, 8 DAF, 12 DAF and 16 DAF siliques, respectively (Figure S2). RT-qPCR analysis of six selected DEGs was consistent with the RNA-seq results (Figure S3, Table S3), which indicated high RNA-seq reliability.

**Figure 4:**
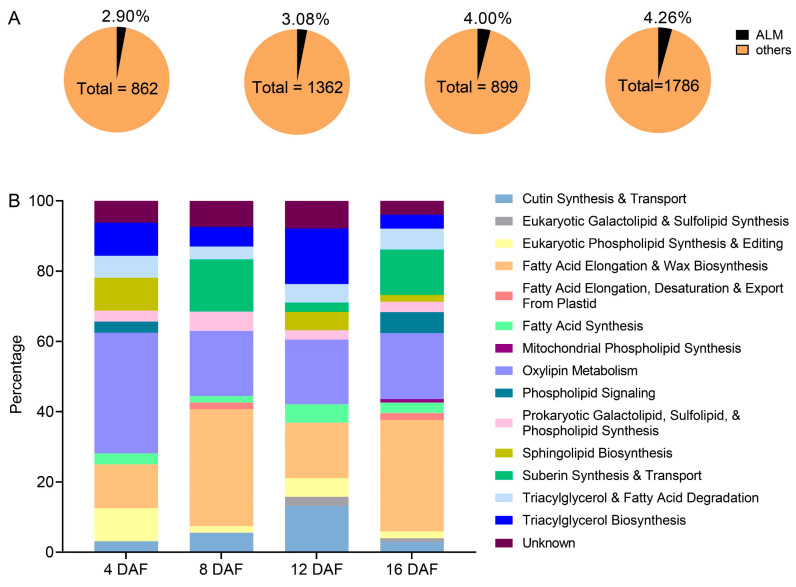
Comparison of gene numbers and pathways involved in acyl-lipid metabolism of siliques at four different developmental stages. (**A**) The proportions of identified ALM genes to the total number DEGs of each developmental stage. Total represents the total number of DEG genes. (**B**) Percentage of genes involved in the 16 pathways of ALM in each developmental stage.

In Arabidopsis, 775 genes identified in acyl-lipid metabolism (ALM) engaged in 16 pathways for the synthesis of fatty acid and triacylglycerol, desaturation, storage, degradation and other pathways [3], accounting for 2.71% of the whole encoded proteins in Arabidopsis (Figure S4). The overlapping DEGs contain 25, 42, 36, and 76 ALM genes for 4 DAF, 8 DAF, 12 DAF and 16 DAF, accounting for 2.90%, 3.08%, 4.00% and 4.26%, respectively (Figure 4A). Most of the ALM DEGs are downregulated, especially at 16 DAF, with only 6 ALM DEGs upregulated (Figure S5). “Fatty acid elongation & wax biosynthesis”, “oxylipin metabolism”, “suberin synthesis & transport” pathways account for the largest three proportion at all of the developmental stages (Figure 4B), indicating that most ALM DEGs aggregate in the synthesis of very-long-chain fatty acid derivatives such as wax, suberin, oxylipin.

In our transcriptional profiles, we found several enzymes required for wax and suberin synthesis were downregulated in OE-9 and OE-18 at one or more developmental stages: fatty acyl ω-oxidases (CYP86A1 and CYP86B1), a fatty acyl in-chain hydroxylase (the cytochrome P450 CYP77A6), acyl-activating enzymes (LACS3 and LACS9), sn-2 glycerol-3-phosphate acyltransferases (GPAT2, GPAT3, GPAT4, GPAT5), and ATP-binding cassette transporter G subfamily (ABCG1, ABCG6, ABCG8, ABCG10, ABCG14, ABCG17, ABCG19, ABCG20, ABCG23) (Figure S5). Oxylipins derived from the metabolism of polyunsaturated fatty acids and the lipoxygenase (LOX) pathway is best known as the primary source [17]. It begins with the regio- and stereo-specific incorporation of molecular oxygen at either the 9th- or 13th- carbon of C18:2 or C18:3 and then generated the precursor of JA-12-oxo-phytodienoic acid (OPDA). This process is catalyzed by LOX allene oxide synthase (AOS), allene oxide cyclase (AOC) and OPDA reductase 3 (OPR3) [18]. And these genes were mostly downregulated in OE-9 and OE-18 lines (Figure S5).

### 2.5. KEGG Enrichment Analysis of the Overlapping DEGs of Two Overexpressing Lines Versus Col-0

**Figure 5:**
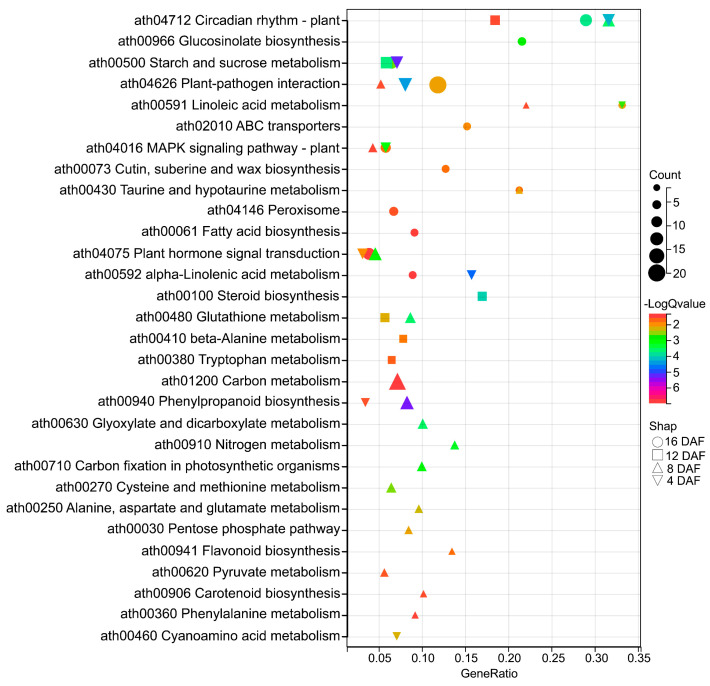
KEGG pathway enrichment results of the overlapping DEGs at four silique development stages of two overexpressing lines versus Col-0. Circle, square, triangular and inverted triangle represent the KEGG pathways enriched by DEGs of 16DAF, 12DAF, 8DAF and 4DAF silique development stages, respectively. The larger the shape, the more genes are enriched in this pathway. The higher the −logQvalue, the higher the significance of the pathway.

KEGG enrichment analysis was conducted on the DEGs identified at four silique developmental stages to obtain information on their potential role in seed oil content regulation. A total of 862, 1362, 899 and 1786 overlapping DEGs were enriched in 11, 22, 8 and 14 KEGG pathways (*p* < 0.05) in the siliques of 4 DAF, 8 DAF, 12 DAF and 16 DAF, respectively (Figure 5, Table S4). Most of the DEGs were enriched in metabolism, including amino acid metabolism (ath00480: glutathione metabolism, ath00250: alanine, aspartate and glutamate metabolism, ath00270: cysteine and methionine metabolism, ath00410: beta-alanine metabolism, ath00380: tryptophan metabolism), carbohydrate metabolism (ath00500: starch and sucrose metabolism, ath00630: glyoxylate and dicarboxylate metabolism, ath00030: pentose phosphate pathway, ath01200: carbon metabolism, ath01210: 2-oxocarboxylic acid metabolism), energy metabolism (ath00910: nitrogen metabolism, ath00710: carbon fixation in photosynthetic organisms), lipid metabolism (ath00591: linoleic acid metabolism, ath00592: alpha-linolenic acid metabolism, ath00073: cutin, suberine and wax biosynthesis, ath00061: fatty acid biosynthesis), and other secondary metabolites (ath00940: phenylpropanoid biosynthesis, ath00941: flavonoid biosynthesis, ath00966: glucosinolate biosynthesis) (Figure 5, Table S4). Notably, most DEGs enriched in lipid metabolism pathways were downregulated in OE-9 and OE-18 compared to Col-0 (Figure S6), which provided a possible explanation for the reduction in seed oil content in the overexpressing lines.

Pathways enriched across multiple developmental stages were considered to have significant implications for understanding the regulatory mechanisms. In present study, five pathways were enriched in three developmental stages, including plant-pathogen interaction (ath04626), plant hormone signal transduction (ath04075), MAPK signaling pathway (ath04016), and linoleic acid metabolism (ath00591) enriched at 4 DAF, 8 DAF and 16 DAF, starch and sucrose metabolism (ath00500) enriched at 4 DAF, 12 DAF and 16 DAF. All DEGs involved in plant-pathogen interaction pathway were downregulated in overexpressing lines compared to Col-0 (Figure S7, Table S4), which might be a possible reason for the reduced resistance of the overexpressing lines to *S. sclerotiorum*. Particularly, the circadian rhythm–plant pathway was enriched across all four silique developmental stages (Figure 5).

### 2.6. Overexpression of AtTLP1b Reprogramed the Circadian Rhythm-Plant Pathway

**Figure 6:**
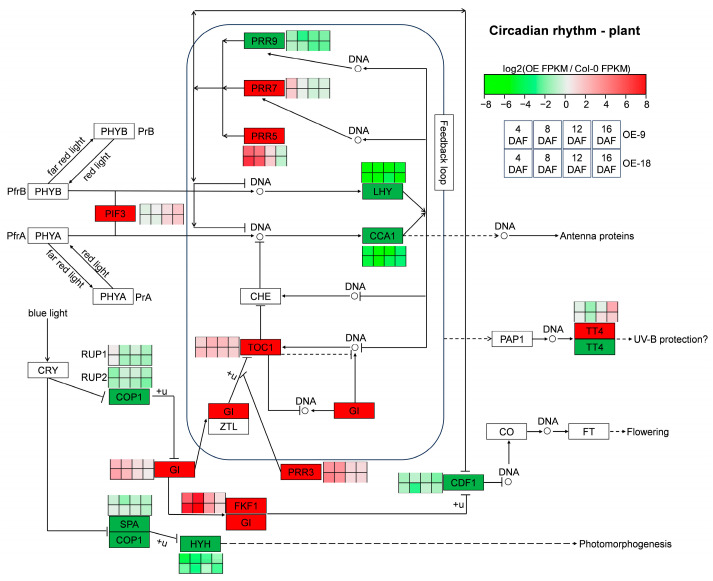
The expression of the overlapping DEGs of two overexpressing lines in circadian rhythm-plant pathway. The red box and green box in the pathway represent that the gene is up-regulated and down-regulated compared to Col-0 at least one developmental stage of siliques. The four squares from left to right in the heatmap represent the silique samples of 4 DAF, 8 DAF, 12 DAF, and 16 DAF, respectively. The four squares at the top of the heatmap belong to the OE-9 line, while the four squares at the bottom belong to the OE-18 line. The solid arrows represent direct effect, and the dotted arrows represent indirect effect.

The circadian rhythm–plant pathway has been demonstrated to play crucial roles in plant development [19]. Results showed that 12, 12, 7, and 11 DEGs were enriched in this pathway in the siliques of 4 DAF, 8 DAF, 12 DAF and 16 DAF, respectively (Table S4), accounting for about half of the circadian rhythm–plant pathway genes. Notably, six genes were differentially expressed among all four developmental stages of siliques. Among these, *LATE ELONGATED HYPOCOTYL* (*LHY*), *CIRCADIAN CLOCK ASSOCIATED 1* (*CCA1*), *PSEUDO-RESPONSE REGULATOR 9* (*PRR9*), *HY5-HOMOLOG* (*HYH*), and *CELL GROWTH DEFECT FACTOR 1* (*CDF1*) were significantly downregulated compared to Col-0, while *TIMING OF CAB EXPRESSION 1* (*TOC1*) was significantly upregulated compared to Col-0 (Figure 6). The expression level of *LHY*, *CCA1* and *TOC1* was also confirmed by RT-qPCR (Figure S3). It was worth noting that the difference in gene expression of *LHY* and *CCA1* between overexpressing lines (OE-9 and OE-18) and Col-0 were the most pronounced (Figure 6), implying that the overexpression of *AtTLP1b* may be closely related to the altered expression level of *LHY* and *CCA1*.

## 3. Discussion

### 3.1. AtTLP1b Is a Potential Target to Enhance Seed Oil Content and Resistance to S. sclerotiorum

Seed oil content is one of the important traits in plant breeding. Mining genes related to oil content and elucidating their genetic mechanisms are of great significance for breeding high oil content varieties and meeting the growing demand for edible oil. In this study, we identified an ER-localized TLP, AtTLP1b, is a novel and effective regulator of oil production in Arabidopsis (Figure 2). Overexpressing and knockout of *AtTLP1b* resulted in 8.75% decreased and 6.1% increased seed oil content in Arabidopsis, respectively. The difference in oil content is caused by changes in C18:1, C18:2, C18:3 and C20:1 between overexpressing and knockout lines (Figure 2D).

We also found that *AtTLP1b* is a negative regulator on *S. sclerotiorum* resistance (Figure 3). Plant breeding is largely constrained by the trade-offs between different agronomic traits, such as the punishment of plant immunity on yield, the dilution effect of plant nutrition and seed quality on yield, and the negative correlation between plant architecture components [20]. Inactivation of the susceptibility gene *Mildew Locus O* (*MLO*) increases durable and broad-spectrum resistance to powdery mildew in plants, but it is also accompanied by growth defects and yield loss [21,22]. The *WSMV resistance gene 1* (*Wsm1*) enhances wheat resistance to wheat streak mosaic virus, but the average yield decreases by 21% [23]. However, we found that *AtTLP1b* could synergistically negatively regulate seed oil content and resistance to *S. sclerotiorum* in Arabidopsis. This makes *AtTLP1b* a potentially promising target for breeding improvement, as plants with high oil content and high resistance could be obtained through gene editing technology alone.

### 3.2. AtTLP1b Regulates Synthesis and Metabolism of Wax, Suberin and Oxylipin Through the Circadian Rhythm–Plant Pathway to Influence Resistance to S. sclerotiorum

Over the past two decades, a number of studies have revealed the role of lipids and lipid metabolites during plant–pathogen interactions. The layer of cuticular waxes and suberin serve as physical barrier that protects plants from environmental assaults, acts as a reservoir of signals to trigger plant defense responses, and even gives cues exploited by pathogens to initiate their infection processes [24]. The KCS gene encodes the β-ketoalionyl-CoA synthetase, which is a rate-limiting enzyme in the synthesis of very-long-chain fatty acids (VLCFAs). Ectopic expression of *MdKCS2* in Arabidopsis increased the content of wax in leaves and stems, altered leaf cuticle permeability, and enhanced plant drought resistance [25]. The R2R3 MYB transcription factor *MdMYB30* modulates plant resistance against pathogens by regulating cuticular wax biosynthesis [26]. Overexpression of *GmLACS2-3* significantly increased suberin content, thereby enhancing drought tolerance [27]. Knockout of *HvMPK3* induced increased suberin accumulation in roots, which may have contributed to the arrested infection by *Fusarium graminearum* [28]. One of the first well-characterized oxylipin pathways was the biosynthesis of the phytohormone JA, which plays a critical role in plant stress responses [3]. The *fad3-2*/*fad7-2*/*fad8* mutant of Arabidopsis is unable to accumulate JA because it is deficient in linolenic acid, the lipid precursor of JA, and exhibits extreme susceptibility to root rot caused by the fungal pathogen *Pythium mastophorum* [29]. Most JA responses are mediated through the coronatine insensitive 1 (COI1) F-box protein. The *coi1-1* mutant exhibits increased susceptibility to the necrotrophic fungi *Alternaria brassicicola*, *Botrytis cinerea*, and *Plectosphaerella cucumerina* [30,31].

In our study, compared to Col-0, most of the ALM DEGs in two overexpressing lines were involved in the synthesis of fatty acid derivatives, particularly surface lipids such as wax, suberin, and oxylipins, which use C16 and C18 as substrate (Figure 4B). The elongation of the C16 and C18 fatty acids into VLCFAs (C20-C36 chains) takes place in the ER [32]. Regarding the localization of AtTLP1b (Figure 1B), it may be part of the elongase complex or interact with its components. We found that most of the enzymes required for wax, suberin synthesis and oxylipin metabolism were downregulated in OE-9 and OE-18 (Figure S5). We hypothesize that this downregulation may result from a reduction in substrates (C18:1, C18:2, C18:3, and C20:1) used for these pathways, ultimately leading to decrease in wax, suberin synthesis, as well as impaired oxylipin metabolism, thereby affecting resistance to *S. sclerotiorum*. Further experimental evidence such as biochemical content and electron microscopy observations are needed to confirm this hypothesis.

Plants’ dependence on sunlight for energy makes their light-driven circadian rhythm/clock a key regulator for coordinating important activities such as development, physiology and metabolism [33,34]. The FA composition exhibits diurnal fluctuations in species such as spinach, cotton, and Arabidopsis [35,36,37,38,39]. Interestingly, in this study, the circadian rhythm-plant pathway was significantly enriched by overlapping DEGs of two overexpressing lines across all four silique developmental stages (Figure 5). The core central feedback loop of the clock in Arabidopsis is regulated by *LHY* and its closely related and functionally redundant homolog *CCA1*, as well as their target *TOC1* [40,41]. The relationship between *LHY*, *CCA1* and *TOC1* is mutually inhibitory [42], which is consistent with our RNA-Seq data (Figure 6 and Figure S2). The *cca1*/*lhy* double mutant exhibits decreased seed oil content and reduced resistance to *Botrytis cinerea*, a pathogen closely related to *S. sclerotiorum* with high evolutionary homology [43,44]. Further research shows that LHY/CCA1 can bind to the promoter of *β-ketoacyl-ACP synthase III* (*KASIII*) and enhance its expression, thereby promoting FA synthesis [41]. These reports indicate that there are interconnections between circadian rhythm/clock, lipid metabolism, and disease resistance. In this study, the expression level of *LHY* and *CCA1* was severely suppressed in the AtTLP1b overexpressing lines (Figure 6 and Figure S3). Therefore, we speculate that *AtTLP1b* may regulate seed oil content and resistance against *S. sclerotiorum* by reprogramming the core circadian feedback loop formed by *CCA1*, *LHY*, and *TOC1*.

## 4. Materials and Methods

### 4.1. Vector Constructs and Plant Transformation

For the p35S::AtTLP1b construct, the coding sequence (CDS) of *AtTLP1b* was cloned into pBin35Sred vector, which contains the red fluorescent protein (DsRed) marker. For CRISPR/Cas9-based knockouts, two 20-bp sgRNA oligonulceotides targeting the conserved thaumatin domain of *AtTLP1b* were inserted into the pCas9-TPC-Red vector which also contains the DsRed marker, to create deletion mutants. The construction method of pCas9-TPC-Red vector followed a previously reported protocol [45]. The primers used to construct these vectors in this study are listed in Table S1. Recombinant plasmids were transformed into the *Agrobacterium tumefaciens* strain GV3101, which was then used for transformation of Arabidopsis Col-0 via the floral dip method [46]. The wild type Col-0 was used as control for both knockout and overexpressing lines. The DsRed marker was used to select transformant seeds [47], and the knockout lines were confirmed by Hi-TOM sequencing [48] and sanger sequencing to verify the target mutations.

### 4.2. Plant Materials and Growth Conditions

For the wild-type Col-0, overexpressing and knockout lines of *AtTLP1b* were grown under long-day conditions (16 h light at 22 °C and 8 h dark at 16 °C) with a light intensity of ca. 120 μmol m^−2^ s^−1^ and relative humidity of 60% in climate-controlled incubators.

### 4.3. RNA Isolation and RT-qPCR Analysis

An SteadyPure Plant RNA Extraction Kit (Accurate Biotechnology (Hunan) Co., Ltd., Changsha, China) was used to isolate total RNA from each frozen sample. First-strand cDNA was synthesized from the RNA by using an All-In-One 5X RT MasterMix (Applied Biological Materials Inc., Vancouver, BC, Canada) according to the manufacturer’s instructions. Primers used for RT-qPCR analysis were designed using Primer 5.0 (Table S1). Each reaction was performed in triplicate with a total volume of 10 μL, containing 0.5 μL of gene-specific primers (10 μM), 2 μL of a 20-fold dilution of cDNA stock solution, 5 μL of BlasTaq 2X qPCR MasterMix (Applied Biological Materials Inc., Vancouver, BC, Canada), and 2 μL of sterile distilled water. The PCR conditions were as follows: 95 °C for 5 min; 40 cycles of 15 s at 95 °C and 45 s at 58 °C; 95 °C for 15 s, 60 °C for 1 min, 95 °C for 15 s. Gene transcript levels were analyzed using RT-qPCR with a Bio-Rad CFX96TM Real-Time qPCR Detection System (Bio-Rad Laboratories, Inc., Hercules, CA, USA). The housekeeping gene *actin8* in Arabidopsis was used as a reference for normalization. Relative gene expression levels were calculated using the 2^−ΔΔCT^ comparative threshold cycle (Ct) method [49]. The Ct values of the triplicate reactions were collected using the Bio-Rad CFX Manager V3.1.1517.0823 software.

### 4.4. Subcellular Localization Analysis

pH7F-woS and pTRAkc-RFP-his6-ERH vectors, provided by Prof. Yonghong Zhou from Southwest University, were used for subcellular localization analysis. The CDS of *AtTLP1b* (stop codon removed) was inserted into the pH7F-woS vector, which contains the CaMV35S promoter and GFP as the reporter gene. The primers used in this experiment are listed in Table S1. The constructed vector plasmids were transformed into *Agrobacterium* GV3101 and co-infiltrated with the ER marker pTRAkc-RFP-his6-ERH into tobacco leaves. Transient expression in tobacco leaves was performed as described in a previous study [50]. Subcellular localization was observed using an LSM780 confocal laser scanning microscope equipped with a ×40/1.2 water immersion objective (Zeiss, Oberkochen, Germany). The excitation wavelengths were 488 nm for GFP and 561 nm for RFP. Images were analyzed using Zen 2.1 software (Carl Zeiss GmbH).

### 4.5. Seed Oil Content and FA Analysis

Seeds from each line were harvested at maturity and then dried in an incubator at 37 °C for three days. Lipids were extracted from the seeds using a previously described method [4]. Seed oil content and fatty acid composition were analyzed by gas chromatography–mass spectrometry (GC-MS) with three biological replicates per line. The GC operating conditions were as described in a previous study [4].

### 4.6. Identification of Resistance to S. sclerotiorum in Transgenic Arabidopsis

The *S. sclerotiorum* strain 1980 was used to assess disease resistance in *AtTLP1b* overexpressing and knockout lines, with Col-0 as the control. Strain 1980 was cultured on potato dextrose agar (PDA) medium to T_3_ generation, and mycelial agar discs (diameter 1 mm) were taken from the active colony edge and inoculated on leaves of 4–5-week-old Arabidopsis. The experiment was repeated three times. Inoculated leaves were cultured in large plastic trays at 22 °C, 95–100% relative humidity in the dark. Lesion area (cm^2^) was measured 24 hpi according to the formula L = π × a × b/4, where “a” defines the longest diameter of the lesion and “b” represents the shortest lesion diameter.

### 4.7. RNA Sequencing and Data Analysis

The siliques at 4 DAF, 8 DAF, 12DAF and 16DAF were collected from Col-0, OE-9 and OE-18, frozen in liquid nitrogen, and stored at −80 °C until RNA extraction. The RNA-seq analysis was performed by BioMarker Technologies (Beijing, China) (http://www.biocloud.net (accessed on 10 October 2020)). Briefly, the cDNA library was constructed and sequenced on Illumina HiSeq 2500 (Illumina, San Diego, CA, USA) platform that yields 100-bp paired-end reads. The raw reads were filtered to obtain high-quality clean reads by removing adaptor sequences, duplicated sequences, reads containing more than 5% “N” (i.e., ambiguous bases in reads), and reads in which more than 50% of the bases showed a Q-value (i.e., Bonferroni-adjusted *p*-value) ≤ 5. The clean reads were aligned to the reference genome of *Arabidopsis thaliana* using the HISAT 2 program [51]. The read counts for each gene were quantified using samtools 1.9 and HTSeq-count 2.0.3, and gene expression levels were normalized as Fragments Per Kilobase Million (FPKM) [52]. The differential expression analysis was performed using the DESeq2 [53]. The threshold determining the significance of differentially expressed genes (DEGs) among multiple tests was set at a false discovery rate (FDR) < 0.01 and |log2 foldchange| ≥ 1 [54]. The Kyoto Encyclopedia of Genes and Genomes (KEGG) enrichment, with a corrected *p*-value < 0.05, was performed using the KOBAS online platform (http://bioinfo.org/kobas/genelist/ (accessed on 18 April 2021)) in order to classify DEGs into the pathway.

### 4.8. Phylogenetic Tree Analysis of the TLP Family in Arabidopsis

Using the “Homology” module of the TAIR database (https://www.arabidopsis.org/ (accessed on 15 January 2025)), 12 proteins belonging to the same TLP family as AtTLP1b were identified. Multiple sequence alignment of TLP family was performed using ClustalW 2 software [55]. The phylogenetic tree of TLP family was constructed using MEGA 7.0.26 software with the Neighbor-Joining method [56]. Bootstrap values (1000 replicates) are shown next to the branches [57]. The evolutionary distances were computed using the Poisson correction method [58] and are in the units of the number of amino acid substitutions per site. Evolutionary analyses were conducted in MEGA 7.0.26 software [59].

## 5. Conclusions

**Figure 7:**
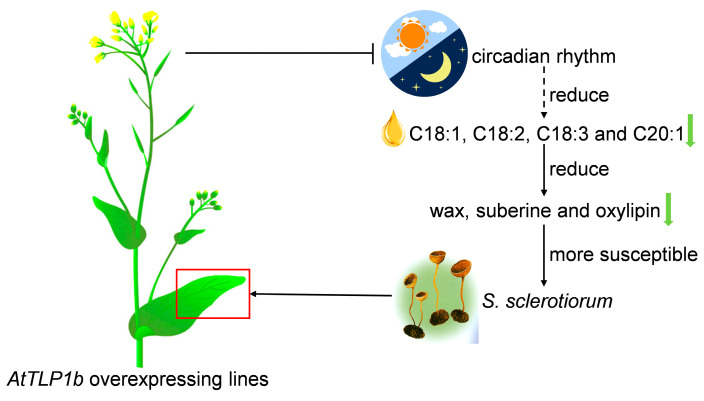
Working model of *AtTLP1b* affects seed oil content and resistance to *S. sclerotiorum* in Arabidopsis. The solid arrows represent direct effect, and the dashed arrow represents possible indirect effect.

In conclusion, we present evidence that *AtTLP1b* is a negative regulator for both seed oil content and resistance to *S. sclerotiorum* in Arabidopsis, and we propose a working model to explain this process (Figure 7). Briefly, the *AtTLP1b* negatively regulates the seed oil content by altering the content of C18:1, C18:2, C18:3 and C20:1 through the circadian rhythm-plant pathway. This regulation leads to a reduced synthesis of wax and suberin, impaired oxylipin metabolism, and ultimately decreased resistance to *S. sclerotiorum*. Therefore, *AtTLP1b* plays a critical role in regulating seed oil content and plant resistance, making it a potential target for gene-based breeding strategies.

## Data Availability

We have deposited the sequencing data into the NCBI database, and it has been assigned the Sequence Read Archive (SRA) accession number PRJNA1134433. All relevant data and plant materials are available from the corresponding authors upon request.

## Supplementary Materials

The following supporting information can be downloaded at: https://www.mdpi.com/article/10.3390/ijms26051930/s1.
