# Supplementary material for: Thaumatin-like Gene TLP1b Confers to Seed Oil Content and Resistance to Sclerotinia sclerotiorum in Arabidopsis
Source: Int J Mol Sci. 2025 Feb 24;26(5):1930. doi: 10.3390/ijms26051930 (PMC11900553; doi:10.3390/ijms26051930)
Supplement: Supplementary file 1 [file ijms-26-01930-s001.zip › Supplementary figures.pdf]

Supplementary information

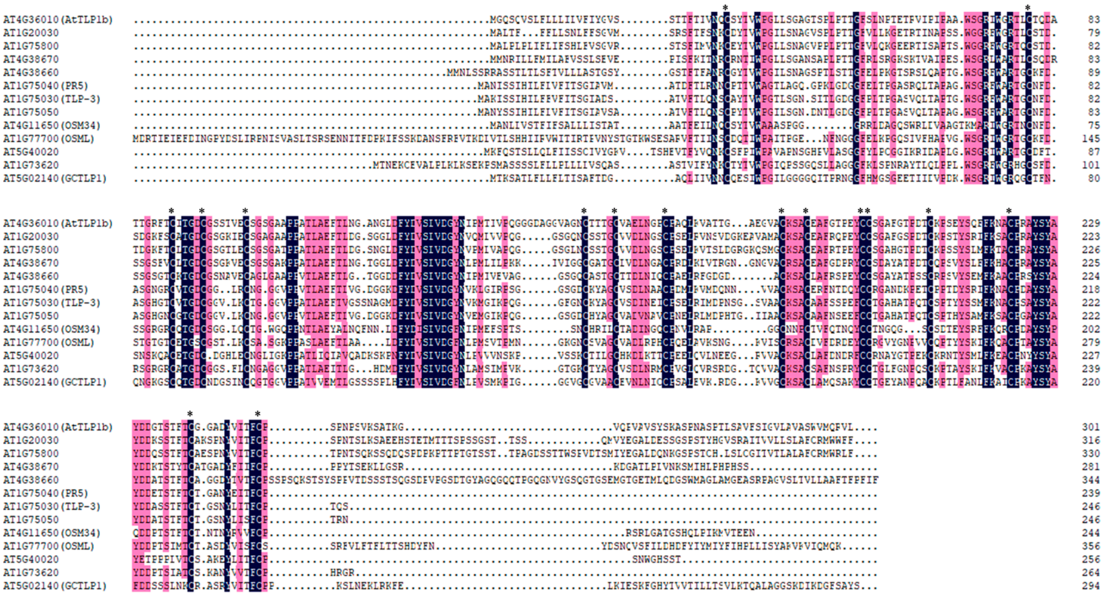

Figure S1. Protein sequence alignment of TLP family in Arabidopsis. Pink represents ≥75% homology level, black represents 100% homology level, and the asterisk represents conserved cysteine residues.

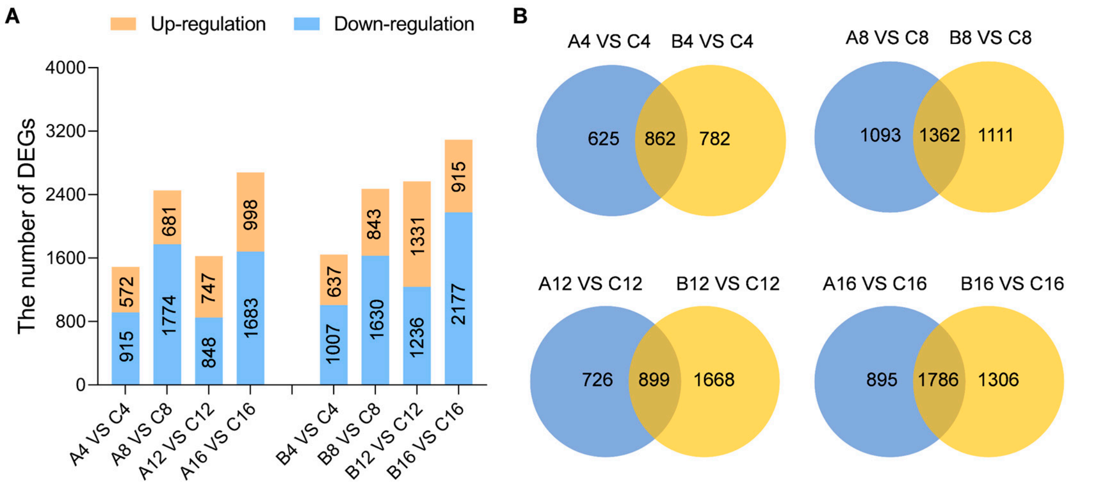

Figure S2. Identification of DEGs in four silique development stages between *AtTLP1b* overexpressing lines and Col-0. A. The number of DEGs in four silique developmental stages of A (OE-9) VS C (Col-0) and B (OE-18) VS C (Col-0). B. The overlapped DEGs of A (OE-9) VS C (Col-0) and B (OE-18) VS C (Col-0) in four silique development stages. 4, 8, 12 and 16 represent siliques of 4DAF, 8DAF, 12DAF and 16DAF respectively.

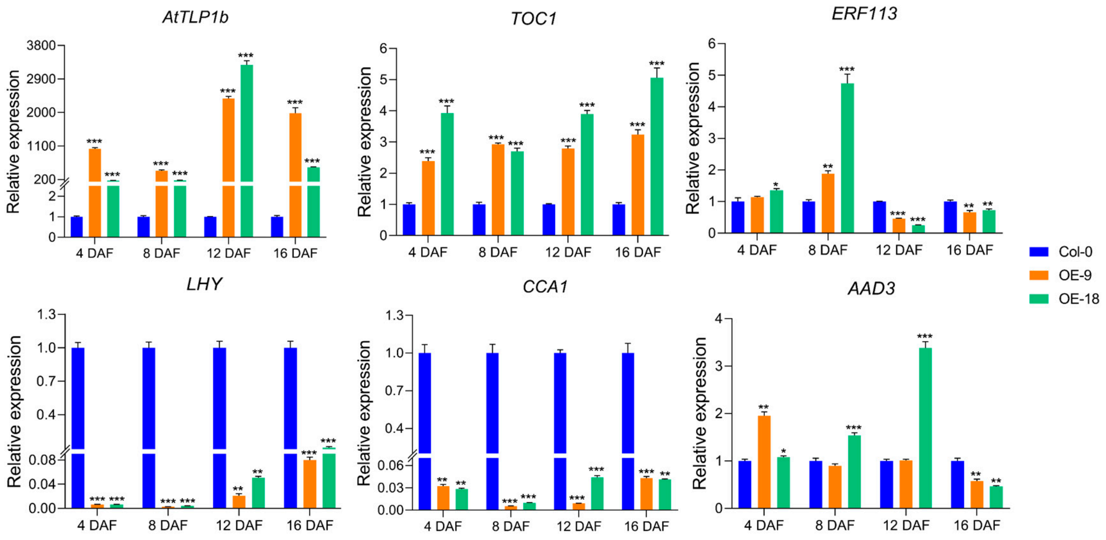

Figure S3. Relative gene expression of six chosen DEGs identified in the RNA-seq experiments. RT-qPCR was performed using cDNA returned from RNA-seq experiments. RT-qPCR values are expressed as the mean ± SD compared to that of the internal control (Actin8). Three technical duplicates were used for RT-qPCR analysis. The asterisk represents significant differences between overexpression lines compared to Col-0 (**P* < 0.01, ***P* < 0.01, ****P* < 0.001. Student's *t*-test).

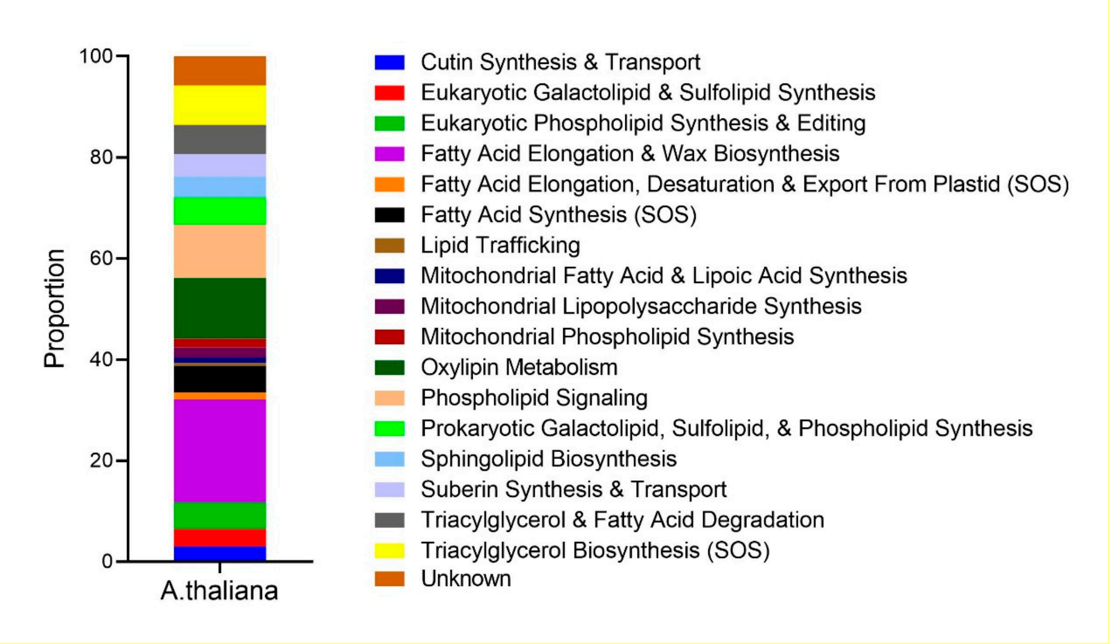

Figure S4. The proportion of 16 pathways engaged by acyl-lipid metabolism (ALM) genes in *A.thaliana*

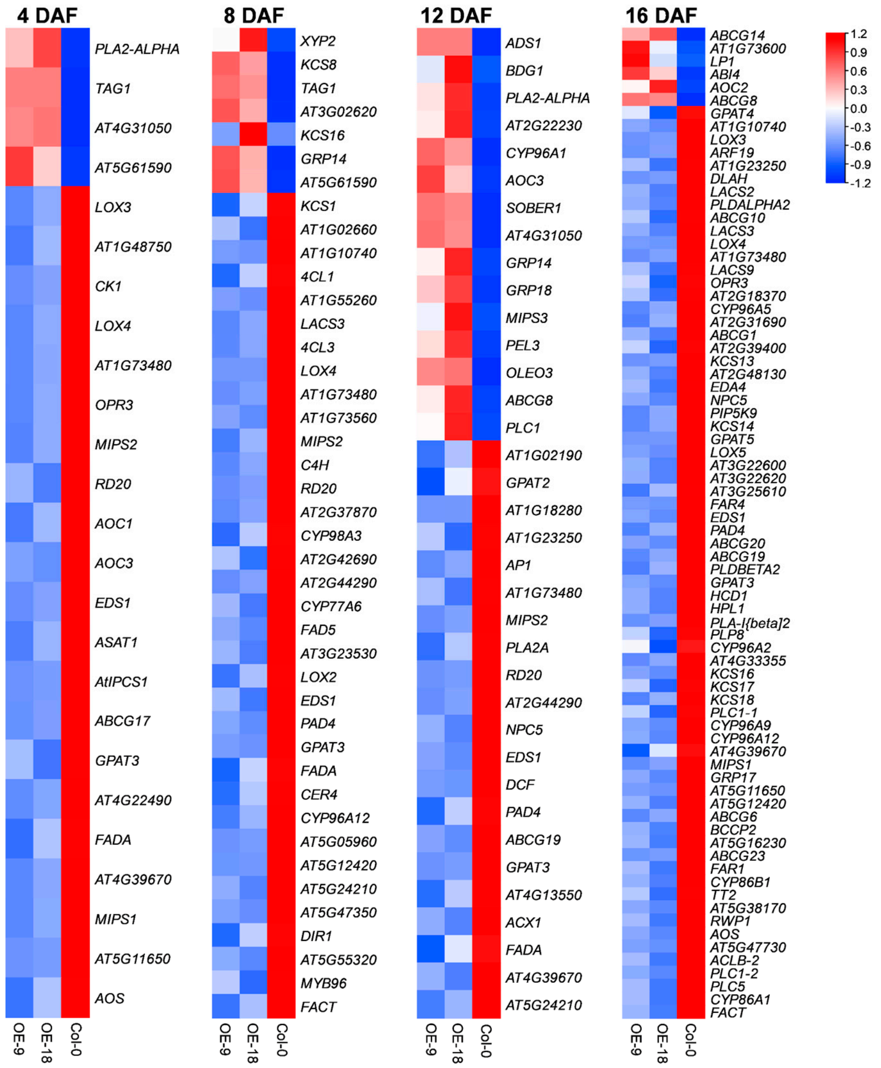

Figure S5. Heatmap of genes involved in ALM at different silique development stages. The expression in the heatmap was normalized. Color ranges from blue to red indicated low to high gene expression.

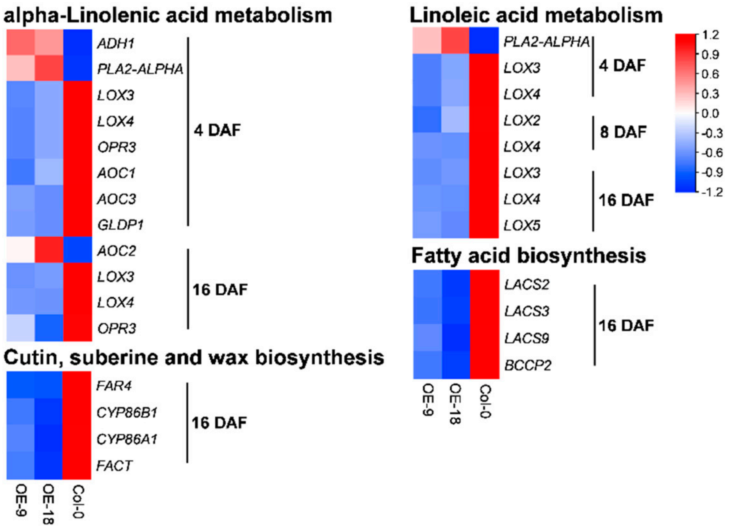

Figure S6. Heatmap of genes involved in lipid metabolism. The expression in the heatmap was normalized. Color ranges from blue to red indicated low to high gene expression.

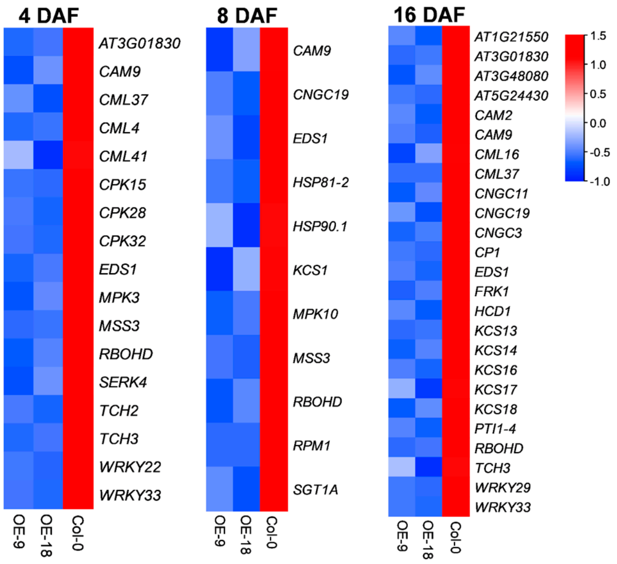

Figure S7. Heatmap of genes in the Plant-pathogen interaction pathway. The expression in the heatmap was normalized. Color ranges from blue to red indicated low to high gene expression.
